# Physiopathology of Lifestyle Interventions in Non-Alcoholic Fatty Liver Disease (NAFLD)

**DOI:** 10.3390/nu12113472

**Published:** 2020-11-12

**Authors:** David Carneros, Guillermo López-Lluch, Matilde Bustos

**Affiliations:** 1 Institute of Biomedicine of Seville (IBIS), Spanish National Research Council (CSIC), University of Seville, Virgen del Rocio University Hospital, 41013 Seville, Spain; dcarneros-ibis@us.es; 2 Centro Andaluz de Biología del Desarrollo (CABD/CSIC/JA), Institute of Health Carlos III (CIBERER), University of Pablo de Olavide, 41013 Sevilla, Spain

**Keywords:** NAFLD, fasting, physical exercise

## Abstract

Non-alcoholic fatty liver disease (NAFLD) is a major health problem, and its prevalence has increased in recent years. Diet and exercise interventions are the first-line treatment options, with weight loss via a hypocaloric diet being the most important therapeutic target in NAFLD. However, most NAFLD patients are not able to achieve such weight loss. Therefore, the requisite is the investigation of other effective therapeutic approaches. This review summarizes research on understanding complex pathophysiology underlying dietary approaches and exercise interventions with the potential to prevent and treat NAFLD.

## 1. Introduction

Nonalcoholic fatty liver disease (NAFLD) is a common disease in one-third of the population in developed countries. NAFLD is associated with metabolic abnormalities including obesity, type 2 diabetes (T2DM), insulin resistance, and cardiovascular disease [1]. NAFLD is a spectrum of liver disease that ranges from simple hepatic steatosis to steatohepatitis (NASH) which is characterized by hepatocyte degeneration (ballooning) and inflammation with or without fibrosis. A small proportion of NASH will further progress to liver cirrhosis and hepatocellular carcinoma [2,3]. 

Diet has a key role in the development of NAFLD. Genetic and positive energy balance have important impacts on the first “hit” and diet composition affects the second "hit" and the severity of NAFLD [4,5,6] emphasizing the criticality of management and control of NAFLD. Several studies have reported that excessive consumption of carbohydrates, especially refined carbohydrates, fats, saturated fats in particular, and protein from meat can cause NAFLD [7,8]. Besides, higher intakes of soft drinks are associated with fatty liver [9].

At present, there is no clear consensus on the pharmacological treatment of NAFLD. In fact, no effective therapeutic agents have been approved for the treatment of the disease. Nevertheless, it is clear that therapeutic approaches should focus on lifestyle modifications. Diet and exercise interventions are the first-line treatment options, with weight loss via a hypocaloric diet being the most important therapeutic target in NAFLD [10]. However, most NAFLD patients are not able to achieve such weight loss. Therefore, the requisite is the investigation of other effective therapeutic approaches. Nutrient composition and caloric intake have been used to devise optimized diets in different stages of NAFLD to control disease progression [11]. Recently, it is recognized that timing and/or frequency of eating meal and fasting (with or without reduced energy intake) can have profound health benefits [12,13]. Nevertheless, more research should be focus on understanding the pathophysiology of the different strategies integrating nutrients, food intake and patterns of frequency of eating meals to provide recommendations for the prevention and treatment of NAFLD.

On the other hand, both aerobic and resistance exercise training in the absence of weight loss has been shown to reduce intrahepatic lipid (IHL) in patients with NAFLD [14]. However, whether exercise training without weight loss can reduce the histological features of NASH and fibrosis remains unknown [15]. Clearly, more studies are required to explore further the molecular and cellular mechanisms involved and to define the optimal volume and intensity of exercise, and whether weight loss is required for histological improvement in NASH and fibrosis.

In this review, we analyze the complex pathophysiology underlying lifestyle interventions for NAFLD. We explore the role of fasting regimens and physical exercise as a first-line treatment option for NAFLD.

## 2. Meal Timing, Frequency and Speed Eating Affect NAFLD

The implication of meal timing and frequency seem to be a factor for metabolic disorders, although state-of-the-art knowledge in NAFLD is scarce and inconclusive. Many studies have provided support the idea that total energy intake at breakfast is inversely associated with weight gain, hypertension and other metabolic alterations [16]. Regarding liver metabolic disease, it was reported that consuming the majority of calories at night is associated with higher risk of NAFLD [17]. A prospective study of NAFLD-free subjects over three years found that individuals who habitually eat before bedtime had double the risk of developing NAFLD [18]. Interestingly, hypercaloric snacking between meals resulted in increased liver fat; however, consuming these same snacks with meals did not cause liver fat accumulation [19]. Recently, it has been demonstrated in mice that timing of food intake might be more important than physical exercise for preventing metabolic disorders, including steatosis [20]. Importantly, in human studies the surprising effectiveness against obesity and NAFLD of fasting without altering caloric intake or source of calories (i.e., time-restricted fasting, TRF) suggests that meal-timing is an important factor to be considered in metabolic disorders in general and metabolic liver disease in particular. Indeed, recent human studies suggest that earlier meal timing associates with improved effectiveness of weight-loss therapy in overweight and obese patients [21]. Taken together, it seems that the timing of eating meals impacts on the development of metabolic alterations; thus, NAFLD could benefit from meal timing intervention. Nevertheless, more human studies are warranted to understand the effect of meal timing and frequency on fatty liver disease.

Eating speed is another factor that can perturb metabolic pathways [22]. The association between eating speed and NAFLD in the general population is controversial. A retrospective cohort study conducted in Japan showed that subjects who habitually ate fast did not have a higher risk of NAFLD [18]. Conversely, another cross-sectional study conducted in a Korean adult population indicated that fast eating is associated with NAFLD [23]. Evidence has also revealed that self-reported eating speed is associated with higher alanine aminotransferase (ALT) activity [24,25], which might lead to NAFLD [1]. In general, eating quickly is a risk for several health outcomes related to metabolic disease [26,27]. Recently, Cao et al. described the association of eating speed and NAFLD, although eating speed was self-reported, indicating that recall bias exists [28]. Mechanisms involved, such as anorexigenic peptides, glucagon-like peptide 1 and peptide tyrosine, produced by the gastrointestinal L cells could cause a weaker anorexigenic gut hormone response, failing to provoke satiety signals [29].

## 3. Strategies and Interventions Altering Meal Frequency and Timing with Fatty Liver Disease: Molecular Mechanisms

Proper nutrition can prevent the onset and progression of NAFLD. Furthermore, altering caloric intake in a nutritionally balanced diet can improve NAFLD [30]. In general, both prolonged reduction in daily caloric intake and periodic fasting cycles can delay the onset of disease and the progression of metabolic disorders [13]. There are four experimental strategies aimed at altering energy intake or the duration of fasting and feeding periods that result in improved aspects with metabolic diseases. (i) classical caloric restriction (CR), in which daily caloric intake is typically decreased by 15% to 40%; (ii) intermittent or periodic full or partial fasting, i.e., a periodic, full- or multiday decrease in food intake; (iii) TRF, which limits daily intake of food to a 4 to 12-hour window; and (iv) fasting-mimicking diets (FMD) that use a strategy by reducing caloric intake and modifying diet composition without necessarily fasting [31].

Restricting the intake of calories induces profound effects, from the transcriptome to the whole physiology and behavior of all animals. CR without malnutrition can be accomplished by chronically reducing energy intake by 15–40% from ad-libitum conditions, while maintaining adequate intake of vitamins and minerals. Energy restriction or CR plays a major role in weight loss and in hepatic fat reduction, so it is considered a central element in nutritional interventions for subjects with NAFLD [32]. Weight loss via CR remains the most viable option in the treatment of NAFLD and fibrosis. The American Association for the Study of Liver Diseases (AASLD) recommends a weight loss of at least 3%–5% of body weight to reduce liver steatosis, while a loss of 7%–10% may be needed to improve fibrosis and the other histological characteristics of NASH [33,34]. Histological improvements have also been observed with as little as 3%–5% weight loss [35,36]. Nevertheless, strategies for NAFLD treatment remain inconclusive and demand further investigation.

**Figure 1:**
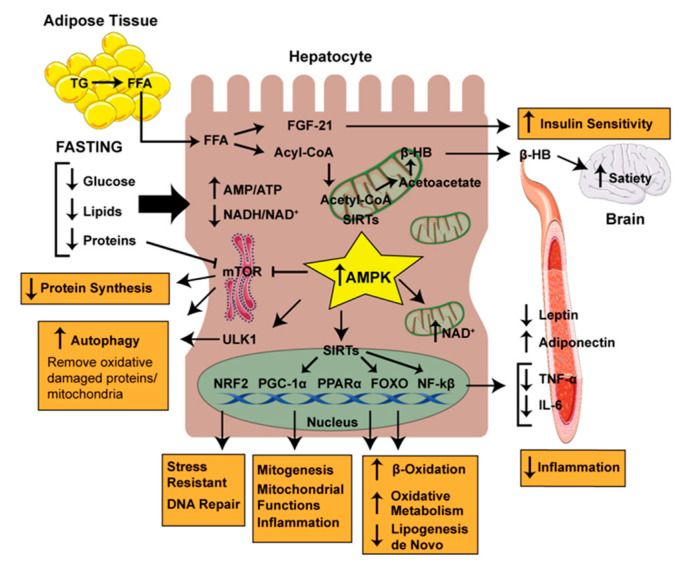
Mechanisms through which fasting might protect against Non-Alcoholic Fatty Liver Disease (NAFLD). Fasting increases lipolysis from adipose tissue releasing free-fatty acids (FFA), which are captured by the hepatocyte. FFA in the hepatocyte stimulates the synthesis of fibroblast growth factor-21 (FGF-21) and alters the ratio of Acetyl-CoA:CoA. Fasting controls the ratios of AMP:ATP and the nicotinamide adenine dinucleotide (NAD+) to NADH. The ratios of these bioenergetic sensors activate AMP kinase (AMPK) and sirtuins (SIRTs). They are main factors in modifications of the transcription factors such as nuclear factor erythroid 2–related factor 2 (NRF2), peroxisome proliferator–activated receptor γ coactivator 1α (PGC-1α), peroxisome proliferator–activated receptor α (PPARα), fork-head box Os (FOXOs) and nuclear factor kappa-B (NF-κβ) (see text). AMPK inhibits mammalian target of rapamycin (mTOR) and activates UNC-51-like kinase-1 (ULK1) resulting in inhibition of protein synthesis and stimulation of autophagy. As a result, fasting favoring stress resistance, mitochondrial biogenesis, and DNA repair decreases lipogenesis de novo, increases β-oxidation and decreases inflammation, all of which support improvements in liver health.

Implementing and sustaining CR have proven to be difficult; thus, intermittent fasting (IF) offers an alternative approach to traditional caloric restriction. Furthermore, chronic CR has been reported to exert adverse effects not only in some mice strains, [37] and safety concerns related to negative outcomes, pointed out within the eating disorders field [38] and related to lack of reserve capacity upon exposure to infection and surgery, have been reported [39,40]. Compelling data may suggest that the benefit of CR and IF goes beyond simple weight loss. A schematic representation of the mediators of the effects of CR/IF on NAFLD response can be seen in Figure 1.

### 3.1. Metabolic Switching: Ketosis

**Figure 2:**
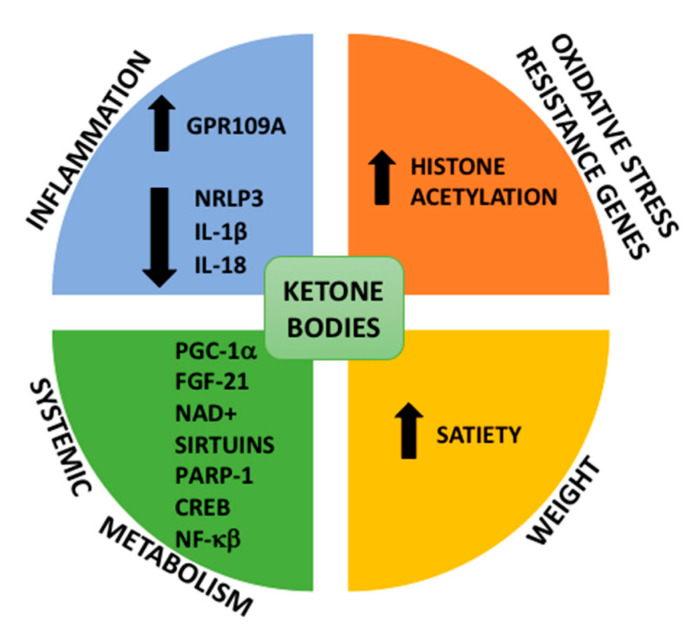
**Role of ketone bodies in the protection of NAFLD (Non-Alcoholic Fatty Liver Disease).** Ketone bodies decrease inflammation by inhibiting NLRP3 and activating GPR109A; increase oxidative stress resistance by histone acetylation; and decrease energy intake by increasing satiety. Ketone bodies have a metabolic role, modifying factors such as peroxisome proliferator–activated receptor γ coactivator 1α (PGC-1α), fibroblast growth factor-21 (FGF-21), nicotinamide adenine dinucleotide (NAD+), sirtuins, Poly(ADP-ribose) polymerase-1 (PARP-1), c-AMP response element-binding (CREB), and nuclear factor kappa-B (NF-κβ).

CR induces profound tissue level changes in metabolism with a generalized shift from carbohydrate to fat metabolism. It is well known that markedly reduced caloric intake on one day or more each week (e.g., a reduction to 500 to 700 calories per day) results in ketogenesis [41]. Ketone bodies (KB) are an alternative source of metabolic energy, especially during states of fasting. KB are generated from fatty acid-derived acetyl-coenzyme A (CoA) through a series of reactions requiring mitochondrial 3-hydroxymethylglutaryl (HMG)-CoA synthase (HMGCS2) expressed in hepatocytes and gut epithelial cells. Acetoacetate (AcAc) represents 25%–50% of the total ketone body pool produced by the liver, with the balance secreted as its reduced form, β-hydroxybutyrate (β-HB), produced by mitochondrial β-HB-dehydrogenase (BDH1) [42,43]. KB are released from hepatocytes into the circulation. β-HB is oxidized to AcAc in the mitochondria of extra-hepatic cells via BDH1, and a CoA moiety is transferred from succinyl-CoA to AcAc via SCOT (succinyl-CoA-oxoacid transferase), which prepares AcAc for terminal oxidation. SCOT is expressed in most of the cells but not in hepatocytes, preventing ketone oxidation in these cells. Compelling evidence suggests a protective role of ketones [44]. β-HB inhibits the NLRP3 inflammasome, decreasing pro-inflammatory cytokines such as IL-1α and IL-18, and fibrosis inducing pyroptosis. It has been reported to have anti-inflammatory effects on macrophages through the G protein coupled receptor GPR109A [45,46]. Moreover, KBs have been proposed to impact in gene transcription through modifications of key histones that serve as regulators of chromatin architecture. Thus, β-HB increased global histone acetylation with subsequent transcriptional changes in mouse models, including genes encoding oxidative stress. β-HB expression may promote oxidative stress resistance [47]. KBs induce satiety although the molecular pathways involved are not very well known [48] leading to decrease of body weight and fat accumulation in the liver. Importantly, KBs regulate the expression of important molecules such as peroxisome proliferator–activated receptor γ coactivator 1α (PGC-1α), fibroblast growth factor 21, nicotinamide adenine dinucleotide (NAD+), sirtuins, poly(adenosine diphosphate [ADP]–ribose) polymerase 1 (PARP1), and ADP ribosyl cyclase (CD38) with clear impact on the systemic metabolism. Finally, β-HB also has signaling functions, including the activation of transcription factors such as cyclic AMP response element–binding protein (CREB) and nuclear factor κβ (NF-κβ) (Figure 2).

In recent years, research has focused on the nutritional induction of ketosis using a ketogenic diet (KD). KD consists of a very low carbohydrate content [49]. There are different types of KD with or without calorie restriction: (i) high fat KDs (HFKD), with a restriction of carbohydrates (< 50 g per day), unrestricted intake of fat, and ad libitum caloric intake; this diet has shown a positive effect on NAFLD both in the short and medium term, independent of calorie and fat intake [50]; (ii) very low calorie KD (VLCKD)(< 800 kcal/day) with carbohydrate intake reduction (< 50 g per day). Most of the studies are in the short term and there are controversial results on the beneficial effects in NAFLD [51,52]. D’Abbondanza and colleagues demonstrated the effectivity of 25-day VLCKD in reducing liver steatosis measured by ultrasonography and liver function tests. Interestingly, they observed that males experienced larger benefits than females in terms of NAFLD improvement [53]. The reduction in carbohydrates in KDs decreases insulin levels with subsequent reduction in lipogenesis in the liver and can lead to a gut microbiota shift limiting oxidative stress and inflammation. Although KDs improved metabolic abnormalities of NAFLD some adverse effects have been reported. Indeed, the metabolic changes closely resembled those seen in starvation with an increased protein catabolism. Increased urinary nitrogen excretion during KD has been shown, [54] with decreased serum concentrations of insulin which stimulates protein synthesis and inhibits proteolysis in skeletal muscle [55]. Some authors refer to safety concerns with KDs (see review [56]); although most studies are of very small sample size and usually of quite short duration.

### 3.2. Stress Resistance, Mitochondrial Biogenesis, Inflammation and Autophagy

IF interventions in humans ameliorate obesity, insulin resistance, dyslipidemia, hypertension, and inflammation [57]. Unfortunately, there are limited data on the effects of fasting protocols on measures of NAFLD. Recently, it has been demonstrated that periodic fasting leads to a clearance of liver fat [58]. Overall, studies clearly show that IF goes beyond body weight loss; however, the mechanisms involved are not clear. 

IF has a myriad of health benefits, including boosting immunity and resistance to stress, improving mitochondria biogenesis and function and decreasing inflammation. IF has also been shown to activate autophagy [59]. Considering these effects, IF may be a possible preventive strategy against NAFLD. In healthy people, IF causes the upregulation of key regulatory proteins of metabolism, DNA repair, and immune system, and results in a serum proteome protective against inflammation and associated lifestyle diseases [60]. Cells respond to IF through an adaptive stress response that leads to increased expression of antioxidant defenses such as superoxide dismutase 1 and catalase in the liver cells [61], antioxidant enzymes NADH-cytochrome b_5_ reductase and NAD(P)H-quinone oxidoreductase 1, heme oxygenase 1, proteins involved in mitochondrial function and biogenesis, and the protein chaperones HSP-70 and GRP-78 [62]. 

Inflammation is increasingly recognized as a contributing factor to the progression of NAFLD and, because excessive energy intake promotes inflammation, it is likely that suppression of inflammation by caloric restriction could play a role in the protection against and progression of NAFLD. Indeed, during Ramadan fasting it has been reported that inflammatory cytokines decrease in patients with NAFLD [63]. Because weight loss may reduce inflammation, it will be important to determine if and how eating patterns modify inflammation independently of weight loss. Mechanistically, fasting and calorie restriction are a natural way to activate the AMP-activated protein kinase (AMPK), an important energy sensor that regulates metabolic homeostasis and decreases inflammation. The activity of AMPK is inhibited by over-nutrition during obesity and NAFLD [64]. A recent study demonstrated that, although the loss of AMPK activity does not affect hepatic lipid accumulation, it substantially exacerbates liver injury and hepatic fibrosis [65,66], both of which could promote the transition from NASH to cirrhosis and HCC. Moreover, activation of AMPK improves symptoms of NASH and therapeutically improves liver injury [66]. AMPK decreases the expression of pro-inflammatory mediators and attenuates inflammation. Mechanistically, AMPK inhibits the nuclear localization of NF-kβ to repress the expression of NF-kβ target genes. Moreover, AMPK activation increases NAD+ levels, leading to the activation of SIRT1. SIRT1 deacetylates NF-kβ RelA/p65 subunit at Lys310 to attenuate its transactivation activity. Other studies have found that multiple downstream transcription factors, including FoxO family proteins and PGC-1α, could be involved in the anti-inflammatory effects of AMPK through regulating gene expression [67]. In addition, AMPK negatively regulates mTOR, and also directly activates ULK1 complex, thereby acting as a positive regulator of autophagy in response to nutrient depletion. IF also upregulates several other autophagy-related proteins such as Atg6, Atg7, Atg8, LC3-II, Beclin1, p62, Sirt1, LAMP2, and ATG101 and thus potentially modulates autophagy. The beneficial role of autophagy in the degradation of lipid droplet in hepatocytes (lipophagy) was described by Singh et al. [68], indicating the important role of autophagy in preventing NAFLD. By contrast, autophagy impairs cellular lipid accumulation, obesity and aging, all of which contribute to the pathogenesis of liver diseases. Therefore, augmentation of liver autophagy is a potential therapeutic approach against liver diseases.

### 3.3. Fasting-Mimicking Diet

In recent years, an alternative nutritional approach has been proposed to mimic the metabolic state that occurs in the human body during fasting, FMD [69]. The goal is to maintain the benefits of fasting, reducing caloric intake and modifying diet composition without fasting. This diet should render it more feasible for patients and increase their compliance. FMD has low calories, provided by plants, herbal team energy bars and supplements, to be gradually implemented in a 5-day cycle each month for 3 months [70]. This diet causes the body to generate energy through gluconeogenesis from non-carbohydrate sources after glycogen store is depleted. In humans, this intervention has been found to ameliorate metabolic disorders, and reduce risk of cardiovascular disease, cancer, cardiovascular disease [70,71] autoimmunity disorders and multiple sclerosis [72]. The majority of findings on the benefits of FMD relate to weight loss. Despite a wealth of proof, solid randomized clinical trials and meta-analyses are still lacking. Furthermore, few studies have reported adverse effects such as hunger, or feeling cold and irritable. In rodents, Wei et al. showed that low protein and low carbohydrate FMD (the diet was administered every other week for a total of 8 weeks) in *ob*/*ob* mice reduced hepatic steatosis and led to the reconstruction of gut microbiota [73]. FMD has been tested in a number of clinical trials. At this moment, there are 20 active study protocols testing FMD in a wide range of different clinical settings, two of them with obesity, but not directly with NAFLD [74]. 

## 4. Exercise and NAFLD 

In addition to diet, the increase of caloric expenditure through exercise can significantly affect the progression of NAFLD. Although controlled exercise programs show different effects, observational studies indicate that non-controlled exercise improve the evolution of NAFLD by decreasing the prevalence rate [75,76]. Interestingly, exercise seems to affect men more than women as indicated by the decrease of the prevalence rate in Japanese people [75]. In a clinical trial using different exercise modalities, low to moderate intensity with high volume of aerobic exercise (50% VO_2_ peak 60 min 4 days/week), high intensity with low volume of aerobic exercise (70% VO_2_ peak, 45 min, 3 days/week) and low to moderate intensity with low volume of aerobic exercise (50% VO_2_ peak, 45 min, 3 days/week), exercise produced a significant but low decrease in liver fat, independently of the type of exercise. The highest effect was found with high intensity or high volume of exercise and showing no impact in the case of low intensity with low volume, indicating that higher energy expenditure is associated with the decrease of fat in liver [77]. The same effect was found with resistance exercise over 8 weeks which produced an important reduction in liver lipids, with increase of lipid oxidation, glucose control and homeostasis without affecting body weight [78]. In a previous study, a program of high intensity and high volume of aerobic exercise (60-70 VO_2_ max, 5 days a week for 30 min), showed normalization of ALT and AST levels in NASH patients, probably indicating reduction of fat in liver [79] and a program of 4 weeks of aerobic cycling exercise also reduced the levels of hepatic triglycerides, although in this study HOMA-IR was not affected [41]. In outpatients with NAFLD plus T2DM, a significant decrease in fatty liver index was found without differences in intensity of the exercise [80]. Resistance exercise also improved many factors in NAFLD patients after 12 weeks of squats and push-ups exercises [81]. In a more recent study, diabetic obese individuals with NAFLD reduced hepatic fat and visceral lipids with both a high-intensity interval and a moderate-intensity and continuous exercise program [82]. This reduction was also associated with the improvement of hepatic damage and inflammation markers in patients with histologically confirmed NAFLD [83]. 

These studies contrast with others that determined the effect of only one bout of exercise on NAFLD markers. Acute exercise did not reduce IHL in NAFLD patients [84] although in this study the determinations were performed after only one bout of exercise and IHL increased after 4 h post-exercise, probably as a physiological response to the metabolic changes induced by the exercise. However, even short-term exercise programs have demonstrated positive effects such as restoring gut hormone regulation in obese adults with NAFLD [85]. 

Many of the studies performed on animals have indicated that aerobic exercise, as with the above indicated diets, improves hepatic lipid metabolism in NAFLD by affecting lipid synthesis, decreasing liver oxidative stress, ameliorating hepatic inflammation and reducing mitochondrial-dependent apoptosis, and inducing autophagy in the liver [86]. The protective effect of exercise against inflammation can be due to the exercise-associated reduction of reactive oxygen species (ROS) overproduction by induction of antioxidant enzymes and anti-inflammatory mediators [87]. 

## 5. Liver and Skeletal Muscle Are Linked in NAFLD 

The nature of association between liver and muscle is not clear and whether alterations of muscle can be a consequence or can aggravate NAFLD remains to be clarified; however, an increased body of evidence links severity and mortality of chronic liver disease (CLD) as results of the progression and impairment of NAFLD, with skeletal muscle depletion. Low muscle mass has been considered a predictor of poor morbidity and mortality occurring in CLD patients [88]. On the other hand, high appendicular skeletal muscle mass, a measure of body muscle content, has been considered as a protective factor for NAFLD in males, although not in females. These gender differences have been associated with different metabolisms and skeletal muscle [89]. 

The relationship between muscle and liver is highlighted by the decrease in muscle mass in most of CLD, and most importantly in cirrhosis. Most of cirrhotic patients show sarcopenia as a common complication [90]. It has been determined that up to 43% of patients with cirrhosis show low muscle mass, 20% show low muscle concurrent with obesity (known as sarcopenic obesity [91]) and 52% show low muscle attenuation [92]. Further, muscle wasting also accompanies liver transplantation indicating a direct effect of liver dysfunction on muscle physiology [93]. Mechanisms directly related to liver status may cause or contribute to muscle depletion [94]. For example, cirrhotic liver causes accelerated starvation leading to protein catabolism and the preferential use of branched chain amino acids for energy supply [95].

Muscle depletion can be a response to overweight or obesity suffered by many NAFLD and NASH patients [96]. The excess weight enforces a constant mechanical pressure that promotes the switch from type I to type II myofibers as has been demonstrated in T2DM patients [97]. Healthy myofibers undergo hypertrophy in response to constant work overload imposed by excess weight [98]. This decrease in oxidative fibers can therefore reduce the capacity to respond to insulin and control plasmatic glucose levels. In obese patients, the loss of type I fibers might result in less efficacy in the utilization of energy substrates. This can induce a vicious cycle in which reduced energy substrate availability leads to myosteatosis and this triggers muscle wasting, impairing insulin response and NAFLD progression. This change in muscle fiber type is, however, not clear in animal studies. In a model of western diet in mice over 16 weeks, significant muscle atrophy was found with increase in low-diameter fibers (probably respiratory) and a reduction in high-diameter fibers (glycolytic) [99].

We can speculate that metabolic disruption in the muscle compartment might also participate in the progression of NAFLD. In a 7-year retrospective study, age-related decrease in muscle mass was found as independent risk factor for NAFLD incidence and, conversely, increase in muscle mass was positively associated with the resolution of existing NAFLD [100]. Although different factors must be considered, the available literature indicates that loss of muscle mass may represent an important factor in NASH pathogenesis [101]. It seems clear that NAFLD and sarcopenia share pathophysiological mechanisms including insulin resistance, inflammation, vitamin D deficiency and lower physical activity [102]. These mechanisms are probably involved in the high risk for NAFLD in individuals with low muscle mass [103]. 

Another important aspect is the relationship of NAFLD to aging and muscle depletion. Muscle mass naturally declines with aging accompanied by a concomitant increase in fat mass. This aging-related sarcopenia seems to be unavoidable and represents a loss of muscle mass caused by exhaustion of muscle stem cell population that impedes regeneration [103], remodeling of muscle fiber type [104] and resistance to anabolic signals [105]. Latest studies have shown that sarcopenia and NAFLD show similar pathophysiological profiles and must be studied in combination in elderly people [106]. In fact, age-related sarcopenia has been recently considered a risk factor for NAFLD and targeting sarcopenia can benefit NAFLD progression [107] probably by reducing hepatic fat. 

### Unbalance in Insulin Growth Factor-1 (IGF-I) Signaling and NAFLD/Sarcopenia Progression

Many studies have consistently shown that NAFLD is associated with impaired insulin action in liver, skeletal muscle and adipose tissue, independently of BMI of the patients [108,109]. Furthermore, relatively small increases in liver fat (approximately 1.5% for liver and around 6% for muscle) induce insulin resistance in these tissues without any further effect or higher increases [110]. This important effect of small increases of fat in the liver severely increase insulin resistance ending in T2DM, and produces further metabolic disorders which also affect normal weight population [111]. On the other hand, the increase of insulin resistance predicts the development of NAFLD by increasing the delivery of free fatty acids from adipose tissue to the liver and increasing de novo lipogenesis [112]. Thus, a small amount of fat in the liver can end in a vicious cycle that promotes the accumulation of fat, the impairment of NAFLD, development of NASH, cirrhosis, and probably hepatocellular carcinoma. 

Muscle and fat are the main organs responding to insulin in the organism. NAFLD-related muscle mass depletion severely increases insulin resistance that can be associated with liver-related signaling. One of these signals is IGF-I. This growth factor is mostly synthesized in the liver and, acting as an anabolic hormone, seems to be relevant for muscle homeostasis. Low secretion of IGF-I from the liver has been associated with NAFLD-related sarcopenia, as recently suggested in a western diet mice model of NAFLD [99]. These animals had reduced muscle strength and lower serum levels of IGF-I in comparison with chow-fed animals, accompanied also by a significant muscle atrophy [99]. In humans, the impairment of the growth hormone (GH)/IGF-I axis has been associated with the risk of the development of sarcopenic obesity together with the accumulation of ectopic fat in the liver [113]. However, it is not clear if the reduction of IGF-I secretion is caused by the damage of liver parenchyma or if the altered GH/IGF-I ratio is the cause of the damage to liver cells [114]. The association of the deficiency of GH with NAFLD and a rapid progression to NASH [115] indicate that this hormone aggravates the evolution of CLD. On the other hand, the age-related decline of this axis, namely somatopause, has been associated with the development of osteoporosis and sarcopenia in the elderly [116].

Although the relationship of the GH/IGF-I axis with NAFLD and sarcopenia are currently under debate, recent mice models fed with a high fat diet for 12 weeks and supplemented with GH or IGF-I have demonstrated that GH and IGF-I supplementation induces significant improvement in both liver steatosis and sarcopenia, indicating that low levels of this factor can represent a severe marker of deterioration in general function of the organism [117]. Further, GH/IGF-I axis unbalance has been considered responsible for the changes in the regulation of protein metabolism in skeletal muscle [116] and in insulin response, since IGF-I stimulates glucose uptake, favoring insulin signaling [118]. Therefore, the control of the ratio between GH and IGF-I is key in the regulation of the insulin response and muscle activity and its unbalance can be related to metabolic unbalance in muscle and also in the liver in NAFLD patients. 

## 6. Exercise Activates Liver-Muscle Signaling Pathways Involved in NAFLD

Physical activity induces a complex system of communication between muscle and liver [119,120]. This communication increases amino acid metabolism, especially branched chain amino acids from muscle and regulates metabolic activities in the liver that induce lipolysis. Exercise not only induces the release of signaling substances such as myokines from muscle [120], but also induces the release of other substances from the liver that control metabolic processes both in the liver and the rest of the organism [119,121]. 

**Figure 3:**
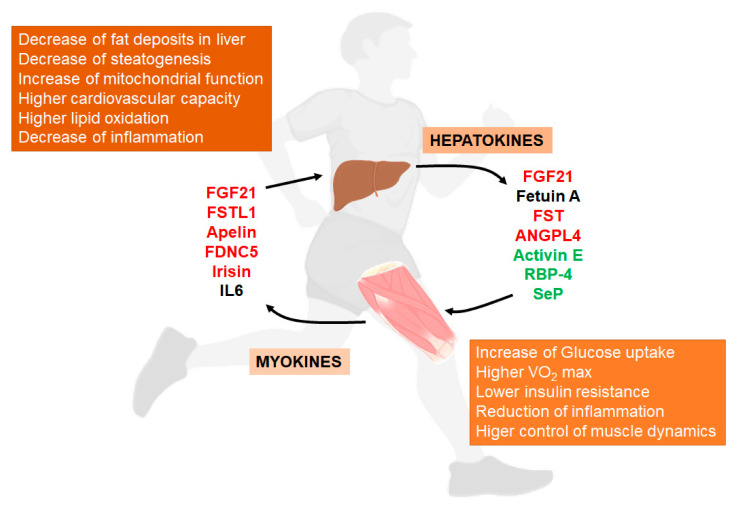
**Liver/muscle crosstalk in NAFLD (Non-Alcoholic Fatty Liver Disease).** Exercise induces the release of several signaling molecules from liver (hepatokines) that, through an endocrine mechanism, improves the physiology of muscle. On the other hand, exercised muscle releases into the circulation other substances called myokines that influence liver physiology, improving the situation caused by NAFLD. Both, hepatokines and myokines reduce the levels of pro-inflammatory markers (see complete names in text). In red, compounds that increase with exercise; in green, compounds that decrease with exercise; in black, compounds with conflicting responses to exercise.

In brief, we summarize some of the main aspects of these mediators and their relationship with NAFLD (Figure 3). 

### 6.1. Hepatokines

Hepatokines are proteins secreted by hepatocytes that influence metabolic processes through autocrine, paracrine and endocrine signaling [122]. NAFLD produces changes in the secretion of these proteins that can increase insulin resistance and induce metabolic dysfunction in many other tissues [123]. Among others, these hepatokines are follistatin (FST), fetuin A and B, retinol-binding protein 4 (RBP4) and selenoprotein P (SeP). NAFLD not only induces changes in the secretion of hepatokines but also produces changes in metabolites, lipids and miRNAs that can alter the metabolism in peripheral tissues including skeletal muscle [109].

#### 6.1.1. Fibroblast Growth Factor 21 

Fibroblast growth factor-21 (FGF-21) is a 24-kDa protein that binds to the classic FGF receptor and the FGF-co-receptor β-klotho [124]. FGF-21 is highly expressed in the liver [125]. FGF-21 is associated with the regulation of energy metabolism, since FGF-21 null mice suffer impairment of glucose metabolism, maladaptation to ketosis and excessive body weight [126]. Furthermore, these mice showed increased hepatic steatosis [127] and inflammation in an IL-17S-TLR4 dependent manner [128], indicating its importance in the impairment of NAFLD. 

Although FGF-21 was initially considered as a myokine [129], some research has demonstrated its release from liver after endurance exercise [130]. Other studies have indicated that FGF-21 production increases in muscle and promotes lipophagy in the liver via an AMPK-dependent pathway [131]. Interestingly, in a rat model of NAFLD, induction of a microRNA, miR-212, decreases the levels of FGF-21 mRNA and protein in liver, and exercise reverses this effect by inhibiting the expression of this microRNA [132]. Metabolic disorders can block the hepatic release of FGF-21 after exercise as has happened in diabetic patients [130]. In fact, the release of FGF-21 is lower in obese patients with hyperinsulinemia compared with healthy subjects [133]. However, this fact must be confirmed by more studies since a recent study showed high levels of FGF-21 levels in NAFLD patients and a decrease after 12 weeks of resistance exercise, in a response attributed to the prevention of the progression of NAFLD [134]. To date, the effect of chronic exercise on levels of FGF-21 is not clear and more research must be performed in order to determine its importance in the inter-organ cross talk [119].

#### 6.1.2. Fetuin A

Fetuin A is a 64-KDa glycoprotein secreted by both, liver and adipose tissue. This hepatokine inhibits insulin signaling and is directly correlated with adiposity in NAFLD patients [135]. Interestingly, the relationship of this hepatokine with the improvement of NAFLD in exercise is not clear since after six months of aerobic exercise and weight loss program, the levels of this hepatokine increased at the same time that glucose metabolism improved [136]. However, a direct relationship between increase in fetuin A and VO_2_max in these patients was found, indicating a relationship of this kepatokine with the improvement of muscle function [136]. On the other hand, a short-term exercise program in obese adults clinically diagnosed with NAFLD produced a decrease in circulating fetuin A levels, along with improved insulin resistance and muscle glucose uptake [137]. The same effect was found in old adults after a supervised exercise training for 12 weeks [138]. Again, the relationship of this hepatokine with physiological improvement in NAFLD patients must be established with more controlled studies. 

#### 6.1.3. Activin and Follistatin (FST) 

Activin E is a member of the TGF-β family [139], considered recently as a hepatokine that is elevated in liver and serum in humans with obesity and NAFLD [140], although many of its functions have been associated with the regulation of adipose tissue [141]. Activin has been associated with the increase of steatosis in liver through the induction of the insulin response [142]; and activation of activin receptors by myostatin and activin also favors inflammation and fibrosis [143]. Interestingly, activin A has also been associated with the increase of atrophy in skeletal muscle linked to NAFLD [144,145]. 

On the other hand, follistatin (FST) is a member of the TGF-β superfamily that acts as antagonist against myostatin and activin A through binding to their receptors and affects the regulation of skeletal muscle growth [146]. This hepatokine is essential for the normal development of muscle since its absence produces insufficient muscle development and skeletal abnormalities in mice [119]. On the other hand, high levels of FST block myostatin/activin action producing antiatrophic effects in muscle. Although FST is also produced in muscle, exercise seems to increase the release of liver FST to plasma [147] in a response attributed to the increase in the glucagon-to-insulin ratio during exercise [130]. In relationship to chronic exercise, resistance training increases the circulating FST in plasma of elderly overweight women [148]. Although recent studies point to the role of FST in NAFLD and other metabolic disorders, little is known about its long-term adaptation to regular exercise and further experiments must be performed in order to understand its relationship with metabolic modifications induced by exercise. 

#### 6.1.4. Retinol-Binding Protein 4 

Considered also as an adipocytokine, RBP4, has been associated with insulin resistance in skeletal muscle. In humans, clinical cross-sectional studies have shown conflicting results indicating a negative correlation between RBP4 levels and insulin sensitivity [149]. In a NAFLD rat model, a 7-week treadmill exercise program was able to reduce RBP4 levels in plasma, although this decrease was associated with fat tissue [150].

In humans, plasma RBP4 levels are high in T2DM, obesity, metabolic syndrome and cardiovascular disease and interventions such as diet, exercise, antidiabetic drugs and hypolipidemic agents decrease their levels [151]. In children, RBP4 levels were high in obese individuals but changes in lifestyle based on exercise were able to decrease these levels at the same time as reducing inflammatory factors in plasma [152]. 

#### 6.1.5. Angiopoietin-Like Protein 4 

Angiopoietin-like protein 4 (ANGPL4) is a glycoprotein of approximately 45–65 kDa secreted by liver and adipose tissue [153]. Although its relevance in metabolic disorders of this hepatokine is not clear, it is well established that the protein regulates lipid metabolism by stimulating lipolysis in adipocytes [154] and inhibiting lipoprotein lipase activity [155]. Studies performed in humans have demonstrated that systemic ANGPLT4 increases during fasting and is secreted from the liver after exercise [156].

The relationship of ANGPL4 with insulin resistance improvement after exercise is not clear. It has been reported that, in obese people, a 6-month program of exercise and diet did not change ANGPTL4 serum levels indicating that other factors contribute to the insulin sensitivity improvement found after this program [156]. However, other studies have shown increased levels of ANGPTL4 after fasting, chronic CR and endurance exercise in a response associated with the increase of plasma free fatty acids levels [153]. The same result was found after a 12-week exercise program or a hypocaloric diet in obese patients indicating a similar response to both exercise and diet [157]. 

#### 6.1.6. Selenoprotein P 

Selenoprotein P (SeP) is a glycoprotein that can be released by liver and adipose tissue and has been shown to contribute to insulin resistance associated with NAFLD [158]. The effects of exercise on this marker are scarce but recent studies indicate that controlled and forced exercise programs reduce the levels of circulating SeP in NAFLD patients [159].

In general, most of the studies performed in NAFLD patients indicate that exercise partially restores the level of hepatokines to those found in healthy patients. However, some studies introduce some discrepancies that must be resolved in order to clearly determine the relationship of these mediators to the progression of the disease and to the regulation introduced by exercise and/or diet. 

### 6.2. Myokines

Acting as a massive endocrine organ, contracting muscle secretes a number of substances known as myokines [160]. To date some of the most well-known are interleukin (IL)-6, IL-10, IL-15, irisin, myostatin, brain derived neurotrophic factor (BDNF), β-amino-isobutyric acid, meteorin-like, leukemia inhibitory factor (LIF); when secreted, they are acidic and rich in cysteine (SARC) [161]. In this review, we will focus on the myokines that have shown a direct influence on the physiology of the liver and on their relationship with NAFLD.

#### 6.2.1. Follistatin-Like 1 and Apelin

Follistin-like 1 (FSTL1) is considered an adipokine or myokine that has been related to insulin resistance in obese and diabetic patients, producing a pro-inflammatory response [162], although other studies have indicated its cardioprotective effect against ischemic injury [163] and it has been shown that FSTL1 levels are reduced in diabetes patients [164]. Apelin has also been associated with adipose tissue but recently it has also been considered as myokine upregulated after exercise and its release has been associated with decrease of fat levels and improvement of cardiovascular capacity [165]. 

FSTL1 [166] and apelin [165] are expressed in myotubes and released after acute exercise. Both have a favorable effect on energy metabolism and rat studies have demonstrated that acute endurance exercise produces significant increases in plasma just after exercise without affecting tissue levels [167]. In humans, acute sprint interval training consisting of four 30-s all-out cycling efforts with 4-min rest periods also produced significant increases in plasma of both myokines [168]. Their role in NAFLD patients has not been studied in depth but we can speculate that these myokines can have an important effect on the reduction of fat and on the improvement of liver activity. 

#### 6.2.2. FNDC5 and Irisin

Irisin is a PGC-1α-induced myokine product of the cleavage of the fibronectin type III domain-containing protein 5 (FNDC5) [169]. Irisin is secreted by contracting skeletal muscle and has been associated with health benefits via changes in metabolism of white adipose tissue [170]. 

Clinical studies have demonstrated that FNDC5 is essential for maintaining metabolic homeostasis and its dysregulation leads to imbalance of systemic metabolism [171]. Independently of its function as precursor or irisin, FNDC5 has been recently shown as relevant for the regulation of diverse upstream and downstream signaling pathways involved in metabolic syndrome [171]. FNDC5 is increased in fatty liver in both mice and humans without affecting plasma levels or irisin [172]. Downregulation of FNDC5 expression resulted in the increase of steatosis and in insulin resistance and higher apoptosis of primary hepatocytes to TNF-α. Probably, the high expression of FNDC5 in hepatocytes in NAFLD can be the consequence of a protective response against steatogenesis through the local release of irisin, or through the activation of downstream signaling molecules that regulate physiological modifications in response to accumulation of fat [171,172]. 

Circulating irisin levels in patients with hepatic steatosis in comparison with controls are confusing. Some studies have shown that irisin levels are lower in obese, NAFLD and NASH patients in comparison with lean controls [173]. Low levels of serum irisin have been also reported in NAFLD, T2DM and NAFLD + T2DM patients in comparison with controls [174], and more recently a significant decrease of plasma irisin together with the adipokines omentin and vaspin have been reported in NAFLD and alcoholic cirrhotic patients [175]. However, other studies have shown that plasma irisin levels are high in NAFLD patients in comparison with controls [176] and the most recent study has shown that plasma of patients with NAFLD contains higher levels of irisin, in direct relationship with the IHL content [177]; further, in HIV patients without diabetes, higher irisin levels were associated with insulin resistance, NAFLD and subclinical atherosclerosis [178]. To add more confusion, another study did not find differences in the levels of irisin between controls and NAFLD patients [179]. It seems clear that the relationship of NAFLD and plasma irisin levels must be resolved in order to understand the physiological relevance of this myokine to NAFLD progression. 

Although the mechanism is not clear, physical activity produces the release of irisin into plasma in an intensity-dependent manner [180]. The release of irisin can be gender dependent affecting more women than men [181]. Interestingly, irisin release after exercise can impair the progression of hepatic fibrosis by regulating the activation, proliferation, migration, contractility and inflammatory cytokine release from hepatic stellate cells [182]. In a recent study, Zhang et al., [183] demonstrated that irisin protects steatotic liver after ischemia/reperfusion in mice through inhibiting ROS production and improving mitochondrial dysfunction. This effect was associated with the binding of irisin to integrin receptors in hepatic cells and activation of kindlin-2, a member of the 4.1-ezrin-ridixin-moesin (FERM) domain family of proteins that regulates many biological functions after interacting with the cytoplasmic tails of β-integrin subunits [184]. However, the role of irisin-dependent kindlin-2 activation is controversial since this protein is considered a biomarker for poor prognosis of liver cancer patients [184] and its deficiency attenuates mouse liver fibrosis and hepatic stellate cells activation [185]. Again, further research is needed in order to understand the putative hepatoprotective effect of irisin in exercised patients. 

#### 6.2.3. Interleukin-6 (IL-6) 

IL-6 is a well-known cytokine associated with liver disease that increases when NAFLD progresses to NASH [186]. It seems that IL-6 can be involved in the inhibition of hepatic autophagy induced by exhaustive physical exercise since IL-6 null mice show reduced levels of markers of autophagy in the liver [187]. However, the role of IL-6 in the exercise effect on NAFLD patients is puzzling, since their release, together with the levels of other cytokines, has been considered a positive effect, inducing anti-inflammatory responses and improving fat metabolism in the liver [188]. In any case, it seems clear that exercise induces the release of IL-6 from muscle. Plasma IL-6 increases exponentially during exercise depending on intensity, duration, muscle mass and endurance capacity [189,190,191]. Voluntary and electrical muscle contractions 19 min twice a week increased IL-6 levels in NAFLD patients in comparison with controls, improving insulin resistance and hepatic steatosis [192]. 

## 7. The Future of Lifestyle Modification in NAFLD

NAFLD is accompanied by a disturbance of the metabolic balance in the whole organism. Lifestyle interventions, including reduced caloric intake, changes in diets, and physical exercise, have been the first-line therapy in efforts to combat NAFLD. Weight loss through changes in diet and exercise are associated with improvement in NAFLD. Nevertheless, there are several challenges to achieving these outcomes: (i) early identification of NAFLD patients is needed to improve patient outcomes; (ii) understanding is needed of the complex pathophysiology underlying the development and progression of NAFLD and its interplay with other metabolic organs; (iii) although regulation of feeding and physical exercise offer strategies to treat NAFLD, maintaining weight loss is challenging. Novel interventions are needed. Efforts to understand the molecular mechanism underlying the effects of exercise and feeding interventions on the liver will have important impacts in the field of NAFLD therapy. In our view, translating the basic scientific findings into fasting- and exercise-mimicking therapies for NAFLD will be made possible by developing pharmaceutical approaches, or by chemical activation of the critical steps elicited during exercise or fasting interventions.
